# Virtual farm tours—Virtual reality glasses and tablets are suitable tools to provide insights into pig husbandry

**DOI:** 10.1371/journal.pone.0261248

**Published:** 2022-01-10

**Authors:** Aurelia Schütz, Katharina Kurz, Gesa Busch

**Affiliations:** Department of Agricultural Economics and Rural Development, University of Goettingen, Goettingen, Lower Saxony, Germany; University of Nicolaus Copernicus in Torun, POLAND

## Abstract

Apart from improving husbandry conditions and animal welfare, there is a clear public demand to increase transparency in agricultural activities. Personal farm tours have shown to be appreciated by citizens but are limited in their impact because of hygiene requirements and accessibility. Virtual farm tours are a promising approach to overcome these limitations but evidence on their perceptions is missing. This study analyzes how a virtual farm tour is perceived by showing participants (n = 17) a 360-degree video of a conventional pig fattening pen on a tablet and via virtual reality (VR) glasses. Semi-structured in-depth interviews were conducted to analyze perceptions and level of immersion and to elicit differences between media devices. Participants’ perception of the pig fattening pen was rather poor and depended on the recording perspective as well as on the media device. However, housing conditions were perceived more positively compared to the image participants had in mind prior to the study, and thus the stable was considered as a rather positive example. Participants described virtual farm tours as suitable tool to improve transparency and information transfer and to gain insights into husbandry conditions. They appreciated the comfortable and entertaining character of both media devices and named various possibilities for implementation. However, VR glasses were favored regarding the higher realistic and entertaining value, while the tablet was considered beneficial in terms of usability. The presentation of video sequences without additional explanations about the farm or the housing conditions were claimed insufficient to give an adequate understanding of the seen content.

## Introduction

Intensive systems in animal production have significantly lost public acceptance and trust over the last decades [1, 2]. Especially pig husbandry has been subject to increasing public criticism in many European countries, including Germany [1–3]. Citizens’ interest in agricultural issues and their demand for more information about agricultural practices is growing continuously [2, 4, 5]. Today, many people, especially in urban environments, lack direct contact with farmers and have only little knowledge of production processes in current farming [6, 7]. One possible approach to increase transparency for these people is to provide visual insights into stables [8–10]. In recent years, farmers have already been intensifying efforts in this respect, for example, by offering guided farm tours or by installing webcams in their stables. Although the use of webcams is generally appreciated and rated positively by citizens, basic criticism regarding general housing conditions, namely available space or slatted floors, is still apparent [11]. Picture perspective has further shown to impact peoples’ perceptions of pig fattening and e.g. space allowance in a pig pen [12]. Webcam pictures from conventional farms combined with informational texts can even lead to a worse rating of pig husbandry [13]. In contrast, on-farm tours represent a way to inform citizens about husbandry conditions and farming practices that are highly appreciated by citizens and even improve perceptions of animal welfare [14, 15]. However, personal farm tours are limited in their impact [16]. Due to biosecurity issues, especially in pig and poultry farming, access to stables is very limited. In addition, citizens need to physically reach the farm, which might be a problem for some, especially when considering that farms are often not accessible via public transport. Further, not all farmers might be willing or capable of offering visits on their farms and starting a dialogue with the (critical) public. New technologies might be an option to overcome these limitations. Virtual farm tours using virtual reality (VR) devices might be a suitable approach to reach a large number of people independent of farm location [17], while largely maintaining the feeling of presence of a real farm visit [18]. In addition, time and effort on the farmers’ side are reduced.

VR is an emerging and promising technology that offers an innovative way for information intake [19]. It can be defined as a highly developed type of human-computer interaction that allows users to interact with the computer in a more natural way compared to standard computer devices [20]. Additionally, VR can be described as immersive and/or interactive, where immersive refers to the sensory level that a system has and interactive to the level of impact the user can have on the simulated content [21–23]. The quality of a VR experience, namely, how real the virtual environment is perceived, highly depends on the level of immersion or rather the feeling of presence, which describes the user’s subjective feeling of ‘being in the virtual environment’ [24]. Experiencing presence can lead to a better understanding of the presented situation and content, compared to simply passively looking at a video on a screen [19]. Furthermore, Bailey et al. [25] and Schöne et al. [26] found that the higher the immersion of a virtual environment, the higher the users’ rememberability of the presented content. Generally, a high level of immersion and active participation can be achieved by using so-called head-mounted displays (e.g., VR glasses) [23, 27].

Today, the use of VR is not limited to the field of gaming anymore, but is becoming more and more important in many different areas, such as tourism, psychology, medicine, military, industry, logistics, or education [23, 28–33]. With regard to the agricultural and food sector, the use of VR has already been investigated in educational or consumer behavior contexts, amongst others. Accordingly, the use of VR technologies has been found to be suitable to simulate physical field trips [34], to train welding skills [35], as well as to gain insights into in-store consumer behavior under controlled lab conditions [36]. VR glasses are also used in agricultural education and training (e.g., to simulate difficult or rare training situations) or to provide visual insights into stables at agricultural fairs, as, for example, the Chamber of Agriculture of North Rhine-Westphalia in Germany does. Furthermore, a supermarket in Cologne uses VR glasses and screens at the point of sale to provide consumers with visual insights into livestock stables and with product information (i.e., on husbandry conditions) [37, 38].

However, to the best of our knowledge, there are no scientific studies on people’s perceptions of virtual farm tours. Against this background, our study provides first insights into the perceptions of and attitude towards virtual stable tours in pig husbandry. Using an explorative qualitative approach, 17 students at the local University were surveyed with semi-structured in-depth interviews to answer the following research questions:

1. How do participants perceive a pig fattening pen shown in a 360-degree video (1a), and do different recording perspectives influence perception (1b)?
2. Are there differences between a virtual farm tour using VR glasses and tablets?
3. Can virtual farm tours be a suitable tool to adequately increase transparency in pig husbandry?

## Material and methods

### Study design

**Fig 1:**
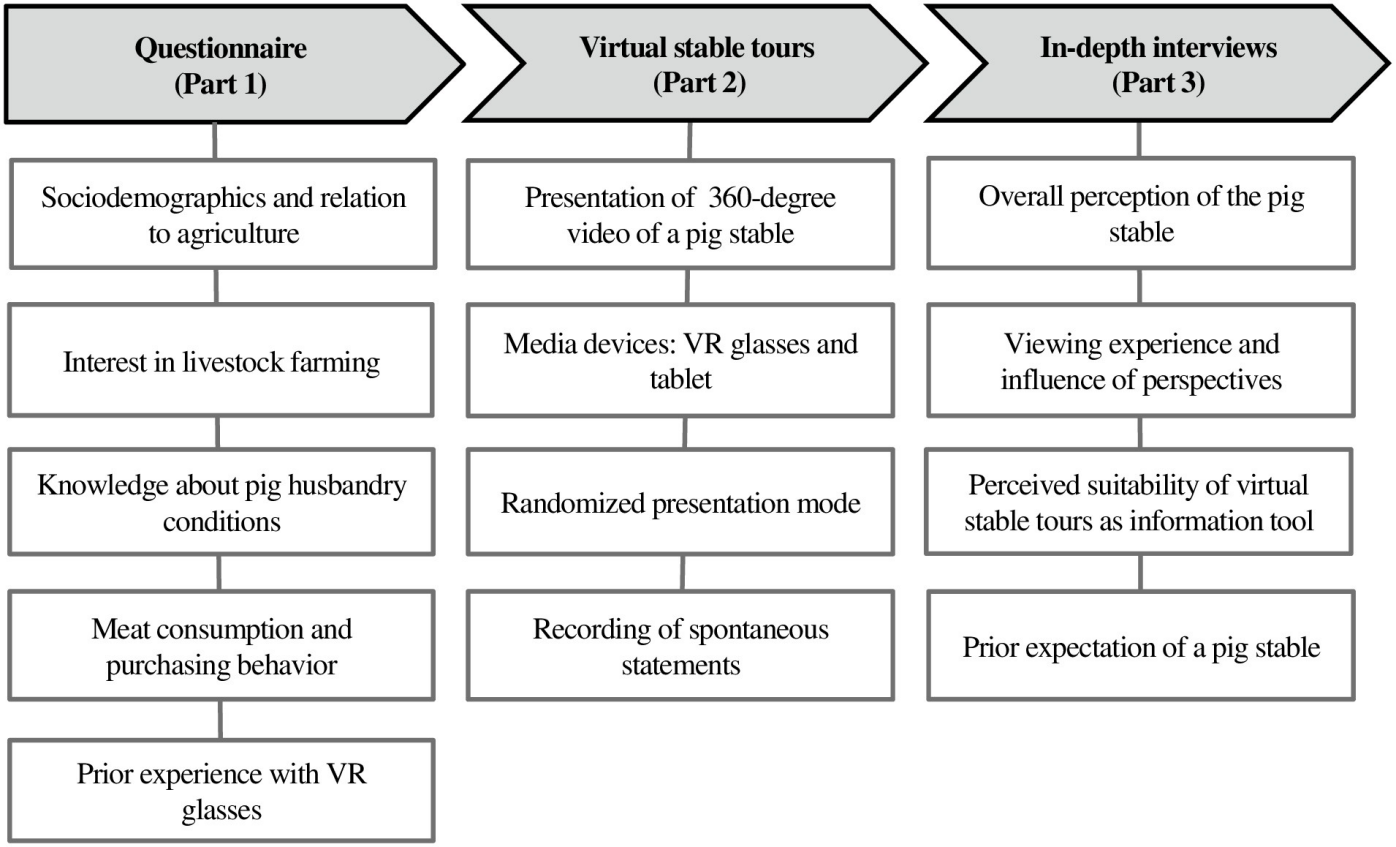
Study design for analyzing perceptions of a virtual stable tour using a questionnaire and in-depth-interview.

Due to the limited availability of research on the perceptions of virtual farm tours via tablets and VR glasses, we decided to use an experimental qualitative research approach consisting of three parts that are displayed in Fig 1. By choosing a qualitative approach, we are able to gain in-depth insights into peoples’ experiences, feelings and perspectives [39–41] when visiting a farm virtually.

#### Questionnaire (part 1)

At the beginning of the experiment, participants were asked to complete a short questionnaire consisting of sociodemographic questions, as well as questions concerning their relation to agriculture, interest in livestock farming, self-perceived knowledge about pig husbandry conditions, meat consumption, purchasing behavior, and prior experience with VR glasses (Fig 1). Apart from dichotomous, single- and multiple-choice questions, five-point Likert scales were used.

#### Virtual farm tours using tablets and VR glasses (part 2)

**Fig 2:**
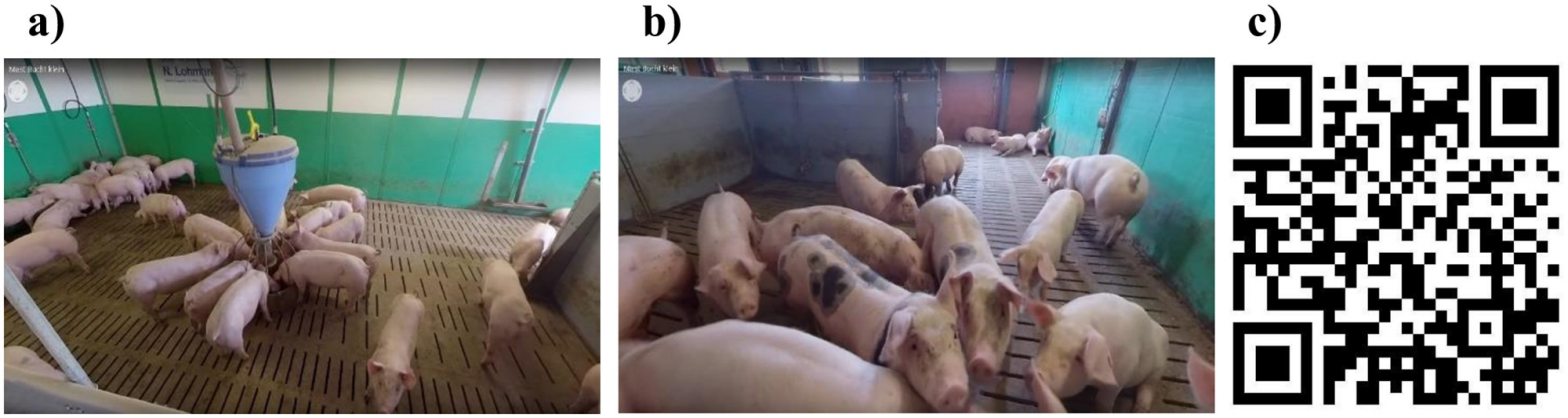
Screenshots from the 360-dregree video used for the virtual stable tours and QR code leading to the video source. Recording perspectives: a) standing human perspective; b) animal perspective, c) QR code leading to the 360-degree video on YouTube. Source: Reprinted from https://www.youtube.com/watch?v=BzeDx5Sxhhw under a CC BY license, with permission from FABRYKANT (Thomas Fabry), original copyright 2020.

In the second part (see Fig 1), participants watched a 360-degree video of a standard pig fattening stable with a fully slatted floor. A screenshot from the video including a QR code leading to the video source can be found in Fig 2. We used a within-subject design in which participants watched the video twice in randomized order: wearing VR glasses and on a tablet. We used this design to improve the comparability of the two media devices. The devices used were a standalone VR headset (Oculus Go VR glasses) and a tablet (Samsung Galaxy Tab 3). The stable shown in the video consisted of a pig fattening pen divided into two sections by a central aisle, but with a passage where pigs weighing 50–60 kilograms were kept. In intensive pig fattening, pigs usually have a starting weight of 25–30 kg and a final weight of 110–125 kg. Thus, the pigs shown in our video were in a fattening phase in which they already had less space to move around due to their weight gain compared to the beginning, but still had more space than at the end of the fattening phase. The video was recorded from a standing human as well as from an animal perspective (Fig 2).

The perspective changed approximately mid-way through the video. The overall length of the video was 3:05 minutes. Participants had a 360-degree panoramic view from a fixed position, which means that they were able to look around in the stable by either moving their head (i.e., when watching the video via VR glasses) or the device (i.e., when watching the video via tablet) up, down, or to the side. Besides 360-degree video images, both media devices reproduced the original sound from the stable. No additional information, such as details regarding the farm, the housing conditions, or the animals, was given. During the virtual stable tour, participants were asked to speak out loud all the thoughts that went through their mind while watching the videos. These spontaneous statements were recorded using a voice recorder.

#### Semi-structured in-depth interviews (part 3)

The two virtual farm tours were followed by a semi-structured in-depth interview, which was based on the three research questions. An overview of the thematic blocks of the interview can be found in Fig 1. First, participants were asked to describe their spontaneous overall perceptions of the pig fattening pen seen on the farm tours via VR glasses and tablet. The second block included questions focusing upon the viewing experience and the potential influence of the two different recording perspectives. The third block dealt with the suitability of virtual farm tours to convey information about husbandry conditions. Finally, participants were asked to describe their expectation of a pig stable prior to the study and whether this idea differed from the pictures seen during the virtual tour. Questions of the second and third block were asked twice for both media devices separately. All interviews were recorded using a voice recorder.

### Pre-tests

Six pre-tests were conducted prior to the main study. As a result, some minor changes were made to the questionnaire and the interview guideline (e.g., reduction and order of questions). Furthermore, it turned out that the video material used for the virtual farm tours needed some adjustments in several aspects. For example, initially the video contained multiple changes of the recording perspective which seemed to be confusing for participants, as well as the fact that the video showed pigs belonging to two different weight classes. Moreover, the initially planned video length (1:45 minutes) was too short, as several participants asked to watch the video a second time. Subsequently, we reduced the number of perspective changes to one, only kept pictures of pigs belonging to one weight class, and increased the total video length.

### Sample and safety precautions due to the COVID-19 pandemic

Data collection took place in May 2020 at the local University. In order to be able to conduct the study during the COVID-19 pandemic, we developed a hygiene safety concept following the official COVID-19 pandemic requirements. The concept was approved by the internal crisis committee of the University. All hygiene regulations and behavioral rules were clearly communicated to all participants in advance and were strictly followed during the entire study procedure. Basic information about study procedure, data privacy, and hygiene measures was given to all participants via email prior to the study in order to keep the timeframe of direct contact between examiner and participant as short as possible. Participants were recruited online through an announcement on the student job portal of the University by giving basic information about the study subject (i.e., that it would be about virtual farm tours via VR glasses and tablet). Each participant received an expense allowance of 10 euros after completing the experiment. A total of 17 students participated. Students with an agricultural background (i.e., agricultural education or those who grew up on a farm) as well as students who followed a vegetarian or vegan diet were excluded from participation. This means that all participants were meat eaters with no professional relation to agriculture. We excluded vegetarians as well as students with professional relation to agriculture from our study, in order to reduce certain group bias. We therefore focused on a more homogeneous group of students with regard to dietary behavior (i.e. at least partial meat consumption) and specific knowledge about the topic (i.e. no expert knowledge about agriculture). By doing so, participants are potential consumers of products originating from the seen stable and not familiar with pig farming.

### Ethic statement

All participants were well informed about the study procedure in advance. The participants provided their written informed consent to participate in this study. Participants had the right to withdraw from the study at any time without giving any reasons. To assure participants’ confidentiality and anonymity, all identifying information was removed from the transcripts, and participants were referred to according to identification codes (i.e. participant numbers P1-P17). A hygiene safety concept following the official COVID-19 pandemic requirements has been developed. The concept was approved by the internal crisis committee of the University (for more detailed information see the section ‘Sample and safety precautions due to the COVID-19 pandemic’ above).

### Data analysis

The questionnaires (part 1) were analyzed by calculating frequencies using IBM SPSS Statistics 26. The interviews as well as spontaneous statements of the participants made during the farm tour were transcribed and analyzed using the text analysis software MAXQDA 2020 by applying structured qualitative content analysis according to Mayring [42]. Based on the established interview guideline, a deductive category application was completed. To establish a coding system, main textual categories were derived first and subsequently more specified subcategories were formed. In addition to each category/subcategory description, the coding system included a definition of each category’s content, a specific coding rule determining aspects of what a text passage/statement had to contain in order to be assigned to a certain category/subcategory as well as an illustrative example (i.e. quotation) for each category/subcategory. If text passages/statements have fitted several categories at the same time, they were assigned multiple times.

The coding was done by two researchers independently, then assignments of text passages were compared, discussed and adjusted. Direct quotations taken from interviews made in the following results section were translated from German into English and marked with the corresponding participant number (i.e. P1-P17)

## Results

### Sample description

In total, 17 students of nine different faculties participated in the study (Table 1). The participation time for the whole experiment was 40 to 60 minutes. Participants were aged between 23 and 34 years. The gender ratio was quite balanced, and more than half of the participants had spent most of their lifetime in urban, highly urban, or extremely urban areas (Table 1).

**Table 1:** Sample characteristics.

Specification	Sample (n = 17)
**Gender**	
Female	8
Male	9
**Age**	
23–25	7
26–28	8
29–31	1
32–34	1
**Faculty affiliation**	
Economics	3
Social sciences	3
Philosophy	3
Law	2
Biology and psychology	2
Physics	1
Business administration	1
Geography	1
German philology	1
**Urbanity of residence**	
Rural (fewer than 5,000 inhabitants)	4
Urban (5,000 to fewer than 20,000 inhabitants)	5
Highly urban (20,000 to fewer than 100,000 inhabitants)	4
Extremely urban (at least 100,000 inhabitants)	4

While thirteen participants stated to be related to agriculture to some extent (i.e., personal contact to farmers or living in close proximity to a farm), the remaining four participants had no relation to agriculture at all. Regarding their interest in livestock farming, ten participants were a little interested, one was very interested, and four persons chose the answer option ‘partly/partly’, whereas only one person was rather not interested, and another was not interested at all. Participants rated their knowledge about pig husbandry as moderate (n = 8), rather low (n = 6), or extremely low (n = 2), and only one participant rated it as rather high. Nine participants stated to eat meat less than once a week or once a week, while the rest reported to eat meat two to three times or four to five times a week. Furthermore, 12 participants answered that it was rather important or very important to them that the animal products they bought came from animal-friendly husbandry, while the remaining five either answered partly/partly, rather unimportant or very unimportant. With regard to previous VR experiences, all but three of the participants knew what VR glasses were, and six of them had even used VR glasses previously. All of them rated their personal VR experience as somewhat to extremely positive.

### Interview results from the virtual farm tours

In the following result section, we only describe categories/subcategories including five or more assigned statements (i.e. number of mentions ≥ 5), which means that the original total number of categories/subcategories and specifications shown in Tables 3–6 is reduced. However, an overview of all categories and subcategories with corresponding number of mentions and participants can be found as (S1 Table).

Table 2 shows a summary of the main results with respect to the three research questions.

**Table 2:** Summary of the main results of the in-depth interviews conducted after the virtual farm tours.

**Research question 1 a, b**	**Perceptions of the pig fattening pen and influence of the recording perspective**	
	Overall	Rather negative perception of the pigsty (e.g. general impression, available space and stocking density) Prevailing rather negative idea of a pigsty/pig husbandry prior to the study The pigsty shown was considered a relatively positive example of a pig stable
	Animal perspective	More negative perception of the pigsty (e.g. general impression, available space and stocking density) compared to the standing human perspective Stronger emotional involvement Higher detail view
	Standing human perspective	Weaker feeling of presence compared to the animal perspective Weaker remembrance of the seen content Improved overview of the pen thanks to a more distanced view
	**Differences between media devices regarding perception, viewing experience and perceived usability**	
**Research question 2**	VR glasses	More negative perception of available space and stocking density compared to the tablet condition Stronger feeling of presence and therewith a higher realistic impression Stronger remembrance of the seen content Pigs appeared huger and/or closer Higher risk of dizziness for some participants
	Tablet	Lower emotional involvement Easier handling because of a higher familiarity with the technical device More distant view of the stable Lower entertaining/innovative value
	**Suitability of virtual farm tours to increase transparency in pig husbandry**	
**Research question 3**	Pros	Suitable tool to enhance the idea of a pigsty and to improve transparency/information transfer Influence on buying/diet behavior (e.g. consuming more meat from animal friendly production) Overall high entertaining/innovative value of virtual stable tours
	Cons	Superficial nature of the stable tour because of missing additional information Doubts about trustworthiness of the presented content Low influence on buying/diet behavior Unfamiliar user experience (e.g. dizziness and feeling of disorientation) Consumers do not want to see real pictures from stables
	Suitable options/locations for implementation	Tool for general advertising or information purposes (e.g. via internet, TV) Self-promotion tool for farmers (e.g. online or even on-farm) Educational institutions (e.g. schools, universities) Point of sale (e.g. traditional supermarkets, farmers’ markets) City centers, specialized fairs, or other bigger events

#### Perceptions of the pig fattening pen and influence of the recording perspective

To describe their general impressions of the pig fattening pen they were shown, participants used more statements with negative rather than positive connotation (Table 3):

P6: “[The view] is very oppressive. I mean, you get quite a spooky feeling here when seeing this”.

P4: “[…] is just still not, not worth living I think. Now, for example, the one in the back has no more desire, oh my god. Well, that’s sad […]”.

**Table 3:** Categories and subcategories established for research question 1a with corresponding number of mentions and participants.

Category	Subcategory	Number of mentions	Number of participants
Perception of the pigsty			
	General impression		
	Positive	13	6
	Negative	31	11
	Available space and stocking density		
	Positive	18	13
	Negative	33	12
	Primary care (neutral)	6	4
	Other housing conditions		
	Positive	22	10
	Negative	30	12
Perception of the pigs			
	Emotional state		
	Positive	23	7
	Negative	5	3
	Activity behavior		
	Positive	6	5
	Negative	22	10
	Neutral	14	9
	Health condition (positive)	6	4
Classification of the pigsty as a positive example		25	12
Previous idea of a pigsty/pig husbandry			
	Negative	23	15
	Neutral	11	8
	No specific idea	8	6
Influence on the previous idea (no)		14	10
Identity of the videos in both conditions (noticed)^1^		30	14
Change of perspective ^2^			
	Noticed	27	16
	Not sure	5	5

^1^Belongs to research question 2;

^2^Belongs to research question 1b.

Similarly, when it comes to a more precise description of housing conditions, participants more frequently referred negatively to available space and stocking density (Table 3):

P14: “Well, they could move, but there was just not enough space“.

P12. “[…] there are a lot of pigs in a confined space […]”.

Also, with regard to other housing conditions participants made more negative statements by criticizing lacking outdoor access or functional or comfortable areas, the bare and boring environment or the level of dirtiness (Table 3). Positively connotated statements rather referred to the previous idea participants had in mind in the case of available space and stocking density or to more basic aspects regarding other housing conditions:

P5: “Well it was still very crowded, it’s not like that, but I imagined it to be more crowded“.

P1: “Looks pretty clean to me, for a pigsty”.

With regard to statements directly relating to the animals themselves, the ratio of negative and positive statements was more balanced (Table 3). Thus, more positive connotated statements were made on the emotional state of the pigs and on the health condition:

P14: “But they actually look quite happy for the fact that they dwell in such a stable. […] They also looked healthy, not overly fattened up […]“.

P12: “[…] Well, I don’t know if they were in a very bad shape, but they didn’t seem scared or anything like that, but rather curious“.

In contrast, participants more often referred negatively to activity behavior:

P6: “Some are laying there apathetically. The animals are running around here under a lot of stress […]“.

P16: “[…] there were some pigs lying in the corners, I don’t know if they were just relaxing or if they were already completely exhausted“.

However, all in all, most of the participants perceived the pig fattening pen as a rather positive example (Table 3): “However, as I said, I think that this is still a relatively good example, it could be much worse, that there are twice as many pigs in a pile, even more crammed together“(P4). This might be related to the fact, that most of them had a rather negative idea of a pigsty/pig husbandry prior to the study (Table 3) and thus perceived the pig fattening pen more positively: “[…] My idea of a conventional stable was even worse, that there are more animals in it. […] that it is even dirtier. And, I don’t know, just such a horrible feeling. […] that the pigs were laying there, half-dead in the corner, well, you’ve seen such things before, it’s all been in the news, where the animals were lying there, really half-dead on the ground. So, I thought it was still okay, but apparently the animals cannot go outside which I consider very borderline. It’s just not a very good life” (P14). However, finally the majority stated, the virtual stable tour did not influence their general image of pig husbandry, neither in a positive nor in a negative way, but rather remained unchanged (Table 3).

Looking at the two different perspectives (i.e., animal or standing human perspective) from which the 360-degree video has been recorded, all but one of the participants noticed and mentioned the change of perspective (Table 3) and results show, that the perspective influenced participants’ perception of the pigsty to some extent. Thus, negative connotated statements regarding the general impression, available space and stocking density and other housing conditions, as well as statements referring to the activity behavior of the pigs, could be rather assigned to the animal perspective (Table 4):

P6: “[There were] two different sequences [perspectives], and in the first one [standing human perspective], I thought, ‘It’s quite a nice life there’ and in the second one [animal perspective], when you were really in the middle of it, you could already see that the animals were very cramped and biting each other […]. […] being part of the action was very frightening for me as a consumer”.

P12: “There is laying an animal in the back corner, and there are some others standing around and stepping on it and stuff, there is another one that is just sitting there […]. […] it is also quite bare […] and relatively boring in that box. […]“.

**Table 4:** Categories and subcategories established for research question 1b with corresponding number of mentions and participants.

		Animal perspective		Standing human perspective	
Category	Subcategory	Number of mentions	Number of participants	Number of mentions	Number of participants
Perception of the pigsty					
	General impression (negative)	5	2	<5	-
	Available space and stocking density (negative)	9	3	<5	-
	Other housing conditions (negative)	9	3	<5	-
Perception of the pigs					
	Emotional state				
	Positive	6	2	<5	-
	Negative	5	3	<5	-
	Activity behavior (negative)	10	4	<5	-
Other perspective dependent aspects					
	Feeling of presence				
	Strong	33	12	<5	-
	Weak	<5	-	5	4
	Remembrance of the seen content (strong)	8	7	<5	-
	Emotional involvement (high)	14	6	<5	-
	Distant view/good overview of the stable	<5	-	20	14
	Detail view (good)	11	5	<5	-
	Pigs look huge and close/room small	5	4	<5	-

With regard to other perspective dependent aspects, the standing human perspective allowed a more distanced view and/or a better overview of the entire stable, whereas in the animal perspective, the room (i.e. stable) appeared smaller and the animals closer and participants had a more detailed view (Table 4): […] So in the standing human perspective I had a better overview of how many animals there were and how big or how small the stable was. When I was with the animals [animal perspective], everything seemed a bit smaller, because everything was closer together and narrower and of course the animals were more around me […] Some things I didn’t notice as much in the standing human perspective as when I was there with the animals [animal perspective], for example, that there were chains hanging […]“(P12).

Furthermore, participants had a much stronger feeling of presence which amongst others was ascribed to the feeling of being on the same level as the pigs standing in the middle of the stable:

P14: “[…] Being more in the middle of the second sequence [perspective] with the VR glasses had a stronger influence compared to the tablet […]. You just had more a feeling of, ‘Okay, I can look around, the pigs are coming up to me’ […].”

P3: “In the second part, where I was standing with the pigs, I had the impression that I was almost being eaten“.

Additionally, participants stated they would best remember the animal perspective in combination with VR glasses and were emotionally stronger involved in the animal perspective.

#### Differences regarding the viewing experience and perceived usability between the two media devices

Most of the participants recognized that they had seen the same pig fattening pen via both media devices, even though some of them were unsure if the video sequences they saw were exactly the same (Table 3): “Well, at the beginning, I thought it was a different one [video] because the ceilings appeared high, but at some point, I recognized certain reactions from the pigs […]” (P1). Furthermore, more statements referring to the change of the recording perspective could be assigned to the VR condition than to the tablet condition (Table 5).

**Table 5:** Categories and subcategories established for research question 2 with corresponding number of mentions and participants.

		VR		Tablet	
Category	Subcategory	Number of mentions	Number of participants	Number of mentions	Number of participants
Change of perspective (noticed)		10	9	8	6
Perception of the pigsty					
	General impression (negative)	<5	-	5	4
	Available space and stocking density				
	Positive	<5	-	5	4
	Negative	9	8	6	4
	Other housing conditions				
	Positive	7	4	6	5
	Negative	6	5	8	6
Perception of the pigs					
	Emotional state (positive)	8	4	8	4
	Activity behavior				
	Negative	6	4	5	3
	Neutral	<5	-	6	4
Other media device depended aspects					
	Feeling of presence				
	Strong	100	16	7	5
	Weak	<5	-	37	14
	Remembrance of the seen content (strong)	18	13	<5	-
	Emotional involvement				
	High	14	6	<5	-
	Low	<5	-	6	5
	Distant view	5	4	21	12
	Detail view (good)	15	7	12	10
	Pigs look huge and close/room small	19	8	<5	-
Usability					
	Dizziness				
	Yes	6	5	<5	-
	No	6	6	<5	-
	Handling				
	Easy	<5	-	8	4
	Hard	8	6	<5	-
	Entertaining/innovative value				
	High	29	16	6	5
	Low	<5	-	7	6

When it comes to the perception of the pigsty, more positive statements related to available space and stocking density could be assigned to the tablet condition, whereas more negative connotated statements were found related to the VR condition. In contrast, regarding the general impression, more negative statements could be ascribed to the tablet condition (Table 5).

With regard to further media device dependent aspects, wearing the VR glasses, participants more often mentioned that the pigs appeared huger and/or closer compared to the tablet condition, whereas in the tablet condition many participants stated to have a more distant view (Table 5):

P7: “[…] the dimensions feel quite different. […] you feel a bit closer to the pigs [wearing the VR glasses]“.

P2: “[…] especially with the virtual reality glasses, the pigs looked very huge and there were a lot of them on the same spot“.

P8: “With the tablet, you were a bit more detached from the scene, you were not directly in the middle of it and you could, I guess, watch it from a further distance […]“.

Furthermore, results show, that the feeling of presence was clearly stronger in the VR condition, where particpants felt like being part of the scene and could better empathize with the situation/the pigs (Table 5):

P14: “You had the feeling that you were standing or sitting in there with the pigs, which was much more intense than with the tablet”.

P8: “You felt a bit like you were in there, a bit more like you were a pig being kept in there, like a fellow sufferer […], and with the tablet you were a bit more distanced”.

In this context, some participants even expressed the desire to interact with the pigs by touching them because the pigs wanted to interact: “[…] I saw them directly around me, like twelve pigs standing around me with huge heads looking at me the whole time, it felt very real, it actually felt as if I could touch them if I wanted to, but it didn’t work, I tried” (P2). Since the entire visual field was covered while wearing the VR glasses, some participants stated to perceive little input from the outside world and were able to be more immersed in the virtual pig farm. Additionally, being able to move the head and look around in the stable while wearing the VR glasses led to a more realistic feeling.

Contrary to the tablet condition participants had a weaker feeling of presence or a much less realistic impression and rather perceived the stable tour as an ordinary video, watching a common television documentary or the news while the real environment was still present (Table 5):

P12: “But I still got the impression that I wasn’t part of the scene, meaning just watching a video. So if ten is very real, well I would say five”.

P2: “I felt much less present there, it felt more like watching a video or a movie […]“.

P11: “[…] you had the feeling that you were in the middle of it, which was not the case with the tablet, where I still realize that I am in this room“.

They did not have the impression that the pigs were aware of their presence but rather of the cameraman: “[…] With the tablet I was much less involved […]. I mean, I had the feeling that the pigs were aware of someone’s presence, I would say of the cameraman’s, but I didn’t have the feeling it was me” (P17).

This weaker feeling of presence was also triggered by the sound, which came exclusively from the front due to the technical features of the tablet device: “[With the VR glasses] the feeling of presence was quite strong […]. Even more than I could have imagined. Not only due to the visual effect but also the sound, which is not that close to the ear with the tablet” (P15).

With regard to the rememberability of the content shown, the VR condition seems to have a longer lasting effect (Table 5):

P7: “Well, the second part in the VR glasses condition is the one I will remember most, I think […]”.

P1:”[…] because it is still something very special, it sticks in your mind a bit more. Watching videos on a tablet or a cell phone is something you experience almost every day […]“.

Furthermore, wearing the VR glasses, participants were more emotionally involved compared to the tablet (Table 5):

P6: “With the VR glasses it was […] much more thrilling, as if you were really part of the scene and somehow affected by it.”

P17: “You also felt more emotionally involved. So there was this brief moment of ‘Oh my God, they’re so cute, you can’t eat them’[…]. It wasn’t really the case with the [tablet] video”.

P1: “It actually felt like you had a certain bond with the animal, you don’t get that on the tablet […]“.

Regarding the usability, participants considered the tablet beneficial in some respect (Table 5). Thus, they found the tablet easier to handle, which was predominantly ascribed to a higher familiarity with the technical device. In contrast, using VR glasses raised some concerns with regard to handling like disorientation or the feeling of insecurity:

P15: “[…] Since I wear glasses, I had some difficulties to fit the VR glasses over them […], and you are not familiar with it, and the tablet is simply more user-friendly because you know how to handle it […].”

P8: “[…] you are a bit disoriented and somehow thinking you would forget the outside world. I did not know at all if I could get up or turn around or would collide with objects around me […]“.

P2: “[…] rather a feeling of insecurity because I could not see where I was standing, where I was walking […]“.

Another potential detriment to the usability of the VR glasses could be a feeling of dizziness, that some participants reported to suffer, whereas for others dizziness was not a problem at all (Table 5).

However, when it comes to the entertaining and innovative value of both media devices, clear benefits were seen for the VR glasses, with many participants stating that they favored the use of the VR glasses over the tablet, simply because they really enjoyed the VR experience, which was fun and new for most of them (Table 5).

P1: „Well, the VR glasses are more fun than the tablet […], you’re just more into it, that’s something special nowadays […] the VR glasses really are more entertaining“.

P12:”Well, I’m just used to the tablet, so I don’t know if I still enjoyed it that much. The VR glasses were something new, I thought they were cool“.

#### Suitability of virtual farm tours to increase transparency in pig husbandry

Most participants stated that if they had the possibility, they would use virtual stable tours to get informed about the housing conditions of farm animals and were convinced that other people would do so as well. Nevertheless, some of them were also skeptical about the potential to reach other people, especially those who are less interested in the topic (Table 6).

**Table 6:** Categories and subcategories established for research question 3 with corresponding number of mentions and participants.

Category	Subcategory	Number of mentions	Number of participants
Usage potential			
	Own interest in use (high)	21	14
	Interest of other people in use		
	High	16	11
	Low	13	11
Pro and contra arguments for virtual stable tours			
	Pro		
	Improve in transparency/information transfer	28	13
	Enhancing the idea of a pigsty/pig husbandry	27	12
	Strong influence on buying/diet behavior	11	8
	High entertaining/innovative value	52	17
	Easy handling	12	6
	Active information intake	5	4
	Contra		
	Lacking of additional information	23	15
	Low credibility	10	5
	Weak influence on buying/diet behavior	11	9
	Unwillingness to face reality	6	5
	High expenditure of time	5	5
	Unfamiliar user experience	21	11
Suitable locations/options for implementation			
	General advertising/information tool	7	5
	Self-promotion for farmers	10	5
	Home use	6	5
	Point of sale	18	12
	Educational institutions	16	10
	City center	7	5
	Events/specialized fairs	10	9

In this context, several pro arguments were mentioned (Table 6). Thus, virtual stable tours were considered a suitable tool to improve transparency and information transfer as well as to enhance the idea of how pigs are kept in livestock production.

P4: "I think [Virtual stable tours] should definitely be used to inform people who do not know it yet, or those, who just have not actively looked for it".

P1: “[…] with such a video or VR glasses, […] you don’t have to read through any texts, and you don’t have to make something up in your head, but you can simply see what the conditions are and then form your own opinion. So I think that this is very suitable".

P7: „Well, now I have a better idea of how pig farming looks like“.

P17: I would say good [suitable] to get a general impression about the life situation […]“.

Some participants even mentioned the potential influence virtual stable tours may have in the sense of encouraging consumers to reflect and change their buying or diet behavior:

P2: “[…] I can also imagine that this would influence me in the choice of which meat I would buy”.

P3: “Maybe you then feel more like buying organic meat instead of this normal cheap meat, because you think okay, maybe the animals are really treated better there”.

Furthermore, participants mentioned the high entertainment and innovative value of the media devices (especially but not only referring to the VR glasses), which was amongst others described as fun, cool, new or unique experience.

P16: “[…] This sounds a little bit harsh, but because of the technology, there is an entertainment factor in there, so you can also walk around and turn around. Maybe more people would go for it rather than for a documentary about factory farming at 11 pm on ZDF.” (ZDF is a German public-service television broadcasting service).

P4: “I actually found the feature quite funny, I’ve never seen it like this before. I’ve never seen it on a tablet in 3D“.

However, when it comes to the usage potential some statements were less optimistic by bringing up several arguments or risks, which may hinder consumers from using virtual table tours (Table 6). First of all, participants were skeptical due to the superficial nature of the stable tour and asked for additional and detailed information on the farm or housing conditions. For many of them it was difficult to put the seen housing system in perspective, e.g. to assess whether the shown stable can be assessed as rather good, bad or average compared to other stables:

P5: “[Additional information] would have been quite cool, for example, just what kind of farm this is, so whether this is normal, what a stable looks like, or whether this is more of an exception, just a general idea of how many pigs live in there, maybe […].”

P1: “[…] How long do they stay in there, and do they have an alternative area to go outside or something like that? Is this just the night stable or are they in there all day? And what kind of farm is this? […] Is it a good farm, is it high-quality meat, or is it the lowest type of farming, just to put it in perspective that way.”

Furthermore, some participants had credibility concerns in the sense that such virtual farm tours could not reflect real housing conditions but rather are glossed over for marketing purposes. Besides, there were doubts about whether a broad implementation would be feasible, because of consumers’ unwillingness to face reality in the sense of “getting to know something which they do not want to know” (P1). Thus, that people might not want to reflect on the rearing conditions of the animals they eat, was considered as a potential barrier to participate in such farm tours: “I think you don’t really want to know what you’re eating, what animal you’re eating, I think most people don’t want the pig directly present when they have a schnitzel in front of them” (P5).

As a further risk, at least with regard to implementation at the point of sale, some participants named the high expenditure of time such stable tours bring along with, or the unfamiliar user experience (e.g. especially dizziness and feeling of disorientation in the case of VR glasses), which may hinder them to use such virtual stable tours at the point of sale. Some participants were also rather skeptical about the potential impact virtual stable visit could have on the buying or dietary behavior:

P2: “I think that this is only for people who are interested in it anyway. […] I don’t think that I will reconsider my buying behavior because of watching this video“.

P3: “After watching videos like this, you might not buy meat for a day or two, but then you’ll forget about it again“.

With regard to suitable locations or rather options for offering/using virtual farm tours, participants had a wide range of ideas (Table 6). Some statements referred to the use for advertising or information purposes via internet or television in general, others were more specific and addressed the potential for farmers to increase transparency and promote their production systems (e.g. online or even on-farm): “[…] farms can use it to enhance their web presence and say ‘look at this, the sausage comes from a happy pig‴ […] (P2).

Apart from that, some participants emphasized the suitability of the own home, where personal media devices could serve to conduct virtual farm tours (e.g. tablet, VR-glasses or 3D Television).

Furthermore, for many participants the point of sale was considered a suitable location (e.g. supermarkets, farmers’ markets), since this is the place were direct decisions about products are made. Some of them even specified the benefits virtual farm tours might have when offered directly at the point of sale: “I could imagine this in any place where meat is sold. There are different quality classes, so if somebody wants to explain the difference between what classes two and three mean, where the difference is, whether the additional price you pay for the meat is worth the difference achieved for the life of the animals. In this form, it could perhaps be established in supermarkets […] so that consumers can be informed about the differences between the various types of husbandry conditions” (P1).

Also educational institutions, particularly schools, were mentioned as users to teach and make children aware about agricultural food production, especially in urban environments: “[…] At school, I would say—well, I am from a very rural area, I’ve seen the inside of a pigsty and a cow stable before, but maybe people who didn’t have the opportunity, for them it would be something new, and they would have the opportunity to do it without much effort […]” (P12).

Offering virtual farm tours in the city center was considered as a promising approach to reach people:

“[…] I mean, there are a lot of initiatives that simply set up stands in the city, and there are already quite a few that have these monitors on the back and front, or some just attach pictures or wear masks, and I think that the incentive to try this out [a virtual farm tour] is relatively high, and if you were to advertise this, it would probably work quite well […]”

(P11).

Finally, specialized fairs or other larger events were mentioned as suitable locations where people go voluntary to actively seeking information (Table 6).

## Discussion

### Perceptions of the pig fattening pen and influence of the recording perspective

The rather negative perception of the pig fattening pen in our study reflects the generally poor public image of intensive pig husbandry systems and the demand for more natural and species-appropriate husbandry conditions [7, 10]. The pig fattening pen used in our study was an example of conventional pig husbandry, with pigs living in a bare environment on fully slatted floors, without any outdoor access. This husbandry system is seen as inappropriate by large parts of the public [43]. Thus, citizens consider outdoor access, natural floor conditions (i.e., straw, grass, mud), enrichment material or objects crucial for improved animal welfare [7, 10, 43, 44]. Therefore, our findings underpin that current conventional pig husbandry systems are not in line with people’s demands and perceptions of suitable husbandry. Even insights into stables do not generally change this fact.

The virtual farm tours did not lead to increased acceptance of the shown pig housing system. This is more or less in line with the studies of Möstl and Hamm [11] and Gauly et al. [13], which found that visual stable insights via webcam pictures or videos did not lead to an improved acceptance of modern pig husbandry, but rather to even lower acceptance (in the case of Gauly et al. [13]). Including additional explanatory information might have had positive effects on the general evaluation of the virtual farm tours in our study – however, depending on framing since the level of animal welfare in conventional husbandry systems is not only publicly criticized but also needs improvement from a scientific point of view [45, 46].

Furthermore, similar to our results, Wernsmann et al. [47] found that different videos showing a conventional pig fattening pen were perceived very poorly in general, with varying stocking densities having a stronger effect on perception than recording perspectives. However, recording perspectives influenced the perception of the pigsty in our study, at least to some extent. This is in line with previous studies, in which pictures and videos of a pig fattening pen recorded from the standing human perspective were rated more positively [12, 48] and left a less confining impression with the viewer [44]. However, pictures from the animal perspective led to a stronger feeling of presence, a higher emotional involvement, a stronger remembrance of the seen content and a more detailed view, especially in the case of VR glasses. Thus, recording perspectives should be taken into consideration when allowing laypersons visual insights into stables.

### Differences regarding the viewing experience and perceived usability between the two media devices

Available space was rated as particularly poor in the VR glasses condition which might be ascribed to the three-dimensional effect created when watching the 360-degree video via VR glasses. This is supported by the participants’ feeling very close to the pigs and some perceived them as oversized, which might have led stocking density appear to be higher. In light of previous research, which found that stocking density and available space per pig are important criteria for laypersons when evaluating husbandry conditions [44], this effect should be taken into consideration when using VR glasses for virtual farm tours. However, VR glasses turned out to be beneficial compared to the tablet with regard to emotional involvement or the feeling of presence. This can be ascribed to differences in the level of immersion and interaction between both media devices [23] and is in line with a study in the field of nature tourism, which found similar differences of VR glasses and tablets [31]. The strong feeling of immersion that the participants had while wearing the VR glasses is particularly remarkable given the fact that they could only look around in the stable from a fixed predefined position instead of freely moving around or switching back and forth between several positions within the stable. However, future research might consider allowing participants to move back and forth between several positions to further improve the viewing experience and thus make the farm tour even more realistic. Apart from that, it might be useful to include a type of opening credits in the video that simulates the walk into the stable. Furthermore, similar to our results, participants in the study of Pasanen et al. [31] were less distracted by events occurring in the outside world, as the entire visual field was covered by the VR glasses. With regard to usability, the tablet was considered to be easier to handle on the first try due to a higher technical familiarity, whereas the physical movements required to look around in the stable were perceived as more comfortable and realistic and the entertaining and innovative value was considered much higher in the case of the VR glasses.

In conclusion, the results demonstrate that VR glasses and tablets differ with regard to the viewing experience and perceived usability. When it comes to the practical implementation of virtual farm tours, both devices might be suitable but for different purposes or locations. It might be easier to implement virtual stable tours via tablets at the point of sale due to easier handling, whereas VR glasses could be more suitable for less stressful situations, such as during school lessons, at specialized fairs or other events at which a technical briefing can be provided. However, since virtual farm tours via VR glasses are perceived as highly realistic, it could be assumed that they would have similar effects to real-life farm tours and would thus be the most suitable alternative to provide real insights into stables, especially when complemented through explanations by a farmer.

### Suitability of virtual farm tours to increase transparency in pig husbandry

Overall, visual insights into livestock stables via VR glasses and tablets were highly appreciated by participants in our study to improve transparency and information transfer in animal production. This is in line with the effects of existing efforts made by farmers, such as providing visual stable insights either in terms of personal farm visits or via webcam pictures or videos that are generally appreciated by citizens [11, 13, 15]. Participants from our sample considered virtual farm tours to be a suitable tool to get a more precise and complete idea of pig husbandry conditions. This approach could thus be used to inform people about prevailing housing conditions. Moreover, virtual farm tours were perceived as an entertaining experience via both media devices, which was considered likely to enhance people’s interest to engage with the topic, especially in case of the VR glasses. Similar effects were found in the field of agricultural education, where VR had an enriching effect on learning processes by increasing students’ general interest in learning contents and facilitating the understanding of different issues [34]. Furthermore, in the context of tourism, the use of VR and 360-degree videos has been shown to lead to a high interest in nature tourism in Finland irrespective of the device (VR glasses or tablet) [31].

Even though virtual farm tours were highly appreciated in our study, it became clear that the presentation of video images without any additional explanation regarding the farm or the specific housing conditions were insufficient and caused a feeling of uncertainty in some participants. These findings are in accordance with earlier studies that have demonstrated the importance of explanatory information when showing laypeople videos or pictures from animal husbandry. Thus, a study of Wildraut et al. [44] found that videos of livestock stables led to feelings of insecurity when laypeople were asked to evaluate housing conditions of fattening pigs without additional information. Furthermore, results from Busch et al. [12] revealed that unexplained animal welfare efforts on pictures (i.e., enrichment objects) were not even recognized by laypeople, and Wille et al. [49] showed positive effects on the perception of informational texts in the context of animal transport. Apart from explanatory information about the farm and the housing conditions, details on the purpose of the video and about the producer were considered valuable to increase the credibility of the presented content in our study. For example, Ermann et al. [15] found that additional explanation by the farmer during personal farm visits led to a higher acceptance of the husbandry system. Similar, personal informational text written by farmers slightly improved the perception of pig husbandry conditions seen on webcam pictures [13]. In this context, it is also interesting to consider that even though the housing system was generally rated negatively in the sense of animal welfare, the specific record of the stable was perceived rather positively by participants (see section 4.1). It is conceivable that this perception is related to the concerns expressed by some participants that virtual farm tours could be used predominantly for marketing purposes and not reflect real housing conditions. Therefore, appropriate and trustful information, also on the sender, seems to be an essential requirement when implementing virtual farm tours.

When it comes to locations or options for offering virtual farm tours, especially educational institutions (e.g. schools in particular) or the point of sale were frequently mentioned as suitable. The point of sale (e.g., supermarkets or farmers’ markets) was thereby considered a suitable location by some, particularly since virtual farm tours might help to make purchasing decisions. In fact, similar approaches can already be observed in practice. For example, a REWE supermarket uses visual insights into livestock stables to inform consumers about products offered in the store via screens and VR glasses [37, 38]. Whether such approaches are perceived solely positively is unanswered, as some participants argued that people might not want to be confronted with living animals and thereby with the question of death when shopping meat. Even though our results indicate an interest in virtual farm tours via VR glasses and tablets, several difficulties with regard to the implementation at the point of sale were encountered. Apart from general concerns (e.g. about the credibility of the presented content or the willingness of consumers to use farm tours or of supermarket retailers to provide them), participants mentioned several difficulties regarding the use of VR glasses (e.g. lack of experience about how to use them, disorientation and insecurity and potential dizziness). Against this background, it seems reasonable that further research should focus on the practical feasibility of using VR glasses in public by conducting studies under real-life conditions (e.g., at the point of sale). In this context, it could further also be considered to include various social groups in order to obtain a broader representation of social attitudes and to identify group-specific differences.

## Supporting information

S1 Table Overview of all categories and subcategories established for research questions 1, 2 and 3 with corresponding number of mentions and participants. (XLSX)
